# Successful Management of Accidental Colchicine Intoxication After Ingestion of *Colchicum autumnale*: A Case Report

**DOI:** 10.3390/toxics14040309

**Published:** 2026-04-03

**Authors:** Zoltán Kovács-Ábrahám, Timea Aczél, Bernadett Lakner, Miklós Ónodi, Csaba Csontos, Márton Németh

**Affiliations:** 1 Department of Anesthesiology and Intensive Therapy, Medical School, University of Pécs, 7624 Pécs, Hungary; csontos.csaba@pte.hu (C.C.); nemeth.marton.ferenc@pte.hu (M.N.); 2 Department of Pharmacology and Pharmacotherapy, Medical School, University of Pécs, 7624 Pécs, Hungary; aczel.timea@pte.hu; 3 Emergency Medicine Department, Szigetvár Hospital, Clinical Centre, University of Pécs, 7900 Szigetvár, Hungary; lakner.bernadett@pte.hu (B.L.); onodi.miklos@pte.hu (M.Ó.)

**Keywords:** colchicine, plant poisoning, toxicology

## Abstract

We report the case of a 58-year-old man who experienced moderate renal and liver impairment after accidental poisoning with *Colchicum autumnale*, which he confused with wild garlic (*Allium ursinum*). *Colchicum autumnale* contains colchicine, a toxic compound that disrupts cell division. The patient received intensive care therapy, intravenous lipid emulsion, vitamin K supplementation, and N-acetylcysteine replacement. After seven days of hospitalisation, he was discharged in good health. This case highlights the importance of patients presenting at the emergency department with uncertain anamnesis, gastrointestinal symptoms or recent consumption of perennial plants before symptom onset, which should raise the suspicion of intoxication. Early diagnosis, organ-specific supportive therapy, and timely initiation of disease-specific therapy are crucial for improving patients’ outcomes.

## 1. Introduction

Colchicine is a tricyclic, lipid-soluble alkaloid derived from *Colchicum autumnale* (autumn crocus), primarily metabolised in the gastrointestinal tract [1]. As a medication, it is used for its anti-inflammatory properties to treat conditions such as gout and familial Mediterranean fever [2]. However, colchicine has an elimination half-life of 9.3–30 h in humans [3] and a narrow therapeutic window; thus, the difference between a therapeutic and a toxic dose is small, making poisoning a significant risk [4]. Colchicine exerts its toxic effects mainly by binding to tubulin, inhibiting microtubule polymerisation. This disruption affects various cellular processes, including cell division, intracellular transport, and the maintenance of cell shape [1].

Colchicine toxicity may result from the accidental harvesting of the *Colchicum autumnale* plant, which can be easily mistaken for wild garlic (*Allium ursinum*) due to their similar appearance, during spring. Several recent cases mainly involve unintentional exposure [5,6,7], including some fatal poisonings caused by multiorgan dysfunction [8,9,10]. Colchicine can be highly toxic even in small doses, with over 0.5 mg/kg potentially being lethal [11]. Poisoning is a serious, potentially life-threatening condition that typically progresses through three distinct stages. Phase one—within 24 h of ingestion—consists predominantly of gastrointestinal symptoms such as severe abdominal cramps, vomiting, and diarrhoea. Delays in recognising symptoms during the initial phase may lead to irreversible damage or death in the later phase. The second phase, occurring 24 h to 7 days after ingestion, involves multiorgan dysfunction—including renal and liver failure, heart failure, bone marrow depression, and shock—with a high risk of mortality. If the patient survives this phase, recovery begins. During this period, alopecia and rebound leukocytosis may appear [11]. Unfortunately, there is no available specific antidote for colchicine poisoning and so treatment is mainly supportive, requiring intensive care. Experimental therapies, such as Fab fragment antibodies, have been investigated but are not yet commercially available [11].

## 2. Case Presentation

**Figure 1:**
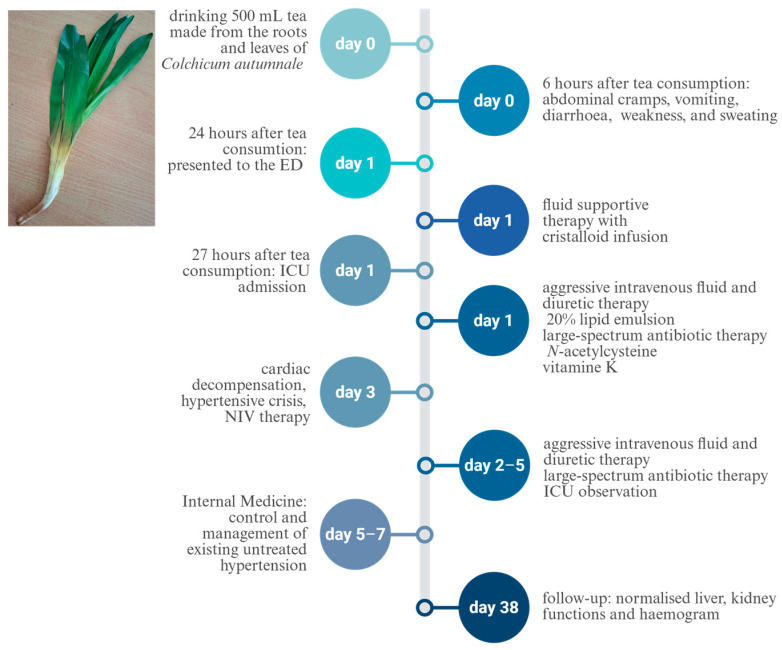
Timetable summarising the patient’s clinical course, diagnostic investigations, treatments, and outcomes. The timeline displays key events from initial presentation to follow-up, including symptom onset, major diagnostic milestones, therapeutic interventions, and significant changes in clinical status. ED: Emergency Department; ICU: Intensive Care Unit; NIV: non-invasive ventilation.

We report a case of poisoning caused by *Colchicum autumnale* (Figure 1) due to confusion with *Allium ursinum* (wild garlic). A 58-year-old man, with a history of untreated chronic hypertension, regular smoking and alcohol consumption, ingested *Colchicum autumnale* by drinking 500 mL of tea made from one root and three leaves of the plant. Six hours after intake, the patient exhibited symptoms such as abdominal and stomach cramps, vomiting, severe diarrhoea, weakness, and sweating. He presented to the emergency department 24 h after consuming the tea, with the aforementioned symptoms, including confusion and slightly slurred speech. He was hemodynamically stable, with a blood pressure of 136/96 mmHg, an SpO_2_ of 99%, and a heart rate of 72 beats per minute. From the admission laboratory parameters, moderately elevated renal function markers—urea and creatinine—should be highlighted, along with an increased transaminase level (AST/GOT) and a mildly elevated INR, which raised the possibility of hepatic injury (Table 1).Blood gas analysis revealed pH 7.34, pCO_2_ 42.9 mmHg, base excess −2.2 mmol/L, HCO_3_^−^ 21.7 mmol/L, and lactate 1.7 mmol/L (Table 2). His abdomen was soft, palpably normal, with no tenderness or abnormal resistance. Cardiopulmonary auscultation was unremarkable. Since the patient admitted to consuming wild garlic tea and brought the plant in question, proper identification was possible. After the botanist identified the plant as *Colchicum autumnale*, the case was referred to the Department of Forensic Toxicology, which recommended only supportive care and multiparameter monitoring in a high-dependency unit, as no specific antidote exists for this condition. Consequently, hydration with balanced crystalloid infusion and transfer to the intensive care unit (ICU) were carried out for the purpose of monitoring potentially severe malignant arrhythmias that may occur in later phases of intoxication, as well as for the observation of other life-threatening toxic complications.

Aggressive intravenous fluid and diuretic therapy were used to promote rapid elimination of the toxic agent (150 mL/h balanced crystalloid, 3 × 20 mg furosemide). Due to the need for high-flow intravenous fluid administration, a triple-lumen central venous catheter was inserted into the patient’s right internal jugular vein.

Intralipid^®^ 20% was administered in accordance with local anaesthetic systemic toxicity (LAST) treatment guidelines [12]: a 100 mL bolus, followed by an additional 100 mL infusion over 20 min. Additionally, a broad-spectrum antibiotic (amoxicillin/clavulanic acid 3 × 1200 mg) was administered intravenously due to an elevated procalcitonin level (1.2 ng/mL) at admission. The patient received a 1200 mg bolus of N-acetylcysteine (NAC), followed by 4000 mg over 4 h and a further 8000 mg over the next 16 h, in accordance with paracetamol intoxication guidelines [13].

On the third day of the patient’s hospital stay, a single episode of severe hypertensive crisis with resulting acute cardiac decompensation occurred, which was managed conservatively using diuretics, morphine, anti-hypertensive agents, and four hours of non-invasive ventilation. The intensive care specialist performed a bedside diagnostic echocardiographic examination during the period of decompensation. The findings were consistent with acute left ventricular failure associated with a hypertensive crisis, with low ejection fraction and dilated left ventricle. This episode lasted approximately 30 min (see Table 2, day 3). The patient did not require any extracorporeal life support. Cardiac Troponin T levels did not show a significant increase, but the NT-proBNP test indicated chronic myocardial frailty (see Table 1, day 3b). Despite aggressive diuretic and intravenous fluid therapy, we aimed to maintain a neutral fluid balance. As shown in the attached fluid balance chart (Table 3), no significant fluid overload was observed until the third day of treatment. However, a contributory role of fluid overload in the development of cardiac decompensation cannot be entirely ruled out.

During the patient’s stay in the intensive care unit, the laboratory parameters evolved as follows: renal function markers (urea and creatinine) normalised; transaminase levels (AST/GOT and ALT/GPT) showed a consistent mild increase; and the INR returned to normal values (Table 1).

After five days, the patient was admitted to the internal medicine department without any complaints. To assess the structural condition of the liver and kidneys, a radiologist performed an abdominal ultrasound examination. Hepatomegaly and a diffuse lesion in the liver parenchyma were confirmed. This abnormality did not impair either the synthesising or detoxifying functions of the liver. At discharge, the coagulation tests (INR) had returned to normal levels. Chronic antihypertensive therapy was established. The patient was discharged home without any complaints, with normalised kidney function but slightly elevated hepatic tests. After one month, he returned for a follow-up examination, where almost all blood parameters had returned to normal, except for leukocytosis. Three months later, during an oral follow-up, the patient, by all accounts, felt well and had no complaints.

## 3. Discussion

*Colchicum autumnale*, commonly known as autumn crocus, is a toxic plant that contains colchicine, a potent mitotic inhibitor with a narrow therapeutic window. Accidental ingestion frequently occurs because these plants resemble edible ones, such as wild garlic (*Allium ursinum*) [11]. Colchicine toxicity, whether pharmaceutical or plant-derived, is a medical emergency with a high risk of multiorgan failure and death if not recognised and treated promptly.

A review of the literature reveals many cases of colchicine poisoning and their treatment. Most of these cases originate from accidental overdose or self-poisoning with suicidal intent. In rarer cases, the source plant was ingested, although such instances are considered unusual. A report, published in 2022, compiled 19 different case reports on colchicine poisoning [3]. Of these, 10 resulted in fatal outcomes despite maximum supportive therapy. Because of this, we believed it was important to publish the case we encountered in our own practice as well.

This case follows the well-described triphasic clinical course outlined in the literature [3]. The first phase is characterised by gastrointestinal symptoms such as vomiting, diarrhoea, and abdominal pain, with initial misdiagnosis as gastroenteritis being possible [14]. In our case, a crucial factor in the successful outcome was the early recognition of *Colchicum autumnale* ingestion, which enabled prompt administration of supportive measures.

Ingestion of more than 0.5 mg to 0.8 mg/kg of colchicine can be fatal [3]. We can only estimate how much colchicine the patient consumed. The colchicine content varies across different parts of the herb and is affected by soil conditions and the harvest season [15,16]. Fresh leaves contain an alkaloid level ranging from 0.15% to 0.4%, while the underground bulbs have an alkaloid content between 0.1% and 0.6% [16,17]. Assuming an 80% extraction from approximately 6 g of leaves and 5 g of root of *Colchicum autumnale*, the tea probably contains at least 10 mg and possibly more than 40 mg of colchicine, greatly exceeding safe or therapeutic doses. The estimated dose of 0.14–0.54 mg/kg falls just below the severe toxicity threshold but within the range associated with serious toxicity.

When colchicine intoxication appears as a differential diagnosis, immediate consultation with the Health Toxicology Information Service, National Center for Public Health, is warranted. Toxicology experts can assist in further diagnosis, provide information about specific treatments, and offer insights into the clinical course of the intoxication. In this case, the strategy was followed, and the actual on-call toxicologist advised supportive therapy. There are no validated specific guidelines for treating acute colchicine poisoning, so management in this case has been based on a recent EM-Review by Wu and Liu [3], following the points below: prevention of colchicine absorption, fluid supportive therapy, intravenous lipid emulsion, forced diuresis, blood purification, extracorporeal life support (ECLS), enhancement of hematopoiesis, antimicrobial therapy, and N-acetylcysteine treatment.

Gastric lavage was not performed because its effectiveness is mainly limited to the first 1–2 h after ingestion. The patient did not receive activated charcoal due to the elapsed time since poisoning, and severe diarrhoea, although its use should be considered, as colchicine undergoes extensive enterohepatic recirculation and clearance can be improved [18]. Aggressive intravenous fluids and diuretic therapy were administered to promote rapid elimination of the toxic agent. Because of the need for high-flow intravenous fluids, a triple-lumen central venous catheter was inserted into the patient’s right internal jugular vein.

Intravenous lipid emulsion (Intralipid^®^ 20%) was administered empirically as rescue therapy. We acknowledge that evidence for its benefit in colchicine poisoning is lacking. For this purpose, Intralipid^®^ 20% was administered in accordance with the American College of Medical Toxicology Position Statement [12].

Unfortunately, there are no consistent findings on the effectiveness of blood purification techniques, such as haemoperfusion and haemodialysis [3]; therefore, this step was not undertaken in the management of this case. Additionally, the patient did not develop multiorgan failure, which would have indicated the need for continuous renal replacement therapy. Furthermore, due to the high volume of distribution of the substance, renal replacement therapy (haemodialysis) has a minor role in the treatment of acute colchicine intoxication [3]. Plasma exchange was also not performed because the patient showed no symptomatic coagulation disorder. Only a slight increase in INR was observed during the initial days of hospitalisation.

No significant changes were observed in blood counts; therefore, neither red blood cell nor platelet transfusion was needed, nor was granulocyte-stimulating growth factor required to enhance haematopoiesis.

Given that the initial inflammatory laboratory parameters were slightly elevated at admission, and considering colchicine intoxication, which is likely to cause immunosuppression with an increased incidence of infection [3], intravenous broad-spectrum antibiotics were commenced. Given the elevated procalcitonin level, there is a strong suspicion of translocation associated with gastrointestinal tract injury. Microbiological samples were unable to confirm a specific infection.

The patient received intravenous N-acetylcysteine (NAC) according to paracetamol intoxication guidelines [13], as no specific NAC protocol exists for colchicine intoxication. NAC therapy is used in the treatment of acetaminophen poisoning, pulmonary fibrosis, and cystic fibrosis, and may have a beneficial effect on colchicine-induced oxidative stress and consequent hepatotoxicity [3,19]. Through its antioxidant properties, NAC protects cells by limiting oxidative stress-induced damage, apoptosis and inflammation [20]. Although there are a few case reports on the use of NAC in colchicine intoxication, based on the hypothesis that NAC may mitigate the inhibitory effects of colchicine on endogenous antioxidants [3,21], more evidence is needed to confirm its effectiveness.

On day three, the patient experienced a hypertensive crisis that led to cardiac decompensation, although there was no significant elevation in troponin levels, but NT-proBNP levels were raised to 2600 pg/mL, which could indicate a chronic heart injury. While untreated high blood pressure and congestive heart failure were diagnosed during hospitalisation, it cannot be ruled out that intoxication may have had some effects on the heart. Given that cardiac toxicity is a major concern in colchicine intoxication [22], it can often result in fatal outcomes. A recent retrospective study concludes that mortality is significantly associated with patients presenting with sinus tachycardia, hypokalaemia, metabolic acidosis, and impaired liver and kidney function [23]. Based on current experience, routine laboratory tests such as creatine kinase, Troponin C, or NT-proBNP may not reliably reflect colchicine-induced cardiac toxicity. Colchicine appears to exert its most significant effects on the cardiac conduction and excitatory systems. In the case presented, we did not observe malignant arrhythmias; however, given the potential for their occurrence, maintaining electrolyte levels in the physiological range and continuous cardiac monitoring are essential in colchicine-poisoned patients to allow prompt detection and management of possible arrhythmias.

## 4. Conclusions

This case highlights the importance of early detection and vigorous supportive care in colchicine poisoning, as well as the dangers of using plants without sufficient botanical knowledge. Many healthcare professionals are unfamiliar with colchicine or *Colchicum autumnale* poisoning and misdiagnosing it as viral gastroenteritis or food poisoning can lead to delayed treatment. It is of the utmost importance to consult the relevant toxicology service regarding possible specific treatments and antidotes. In addition, it is helpful if the emergency physician has experience in toxicology related to cases of poisoning relevant to the local geographic conditions. Although a lack of specific guidelines makes appropriate management challenging, this case report demonstrates that well-structured EM reviews can serve as useful guidelines for physicians in emergency situations.

## Figures and Tables

**Table 1:** Laboratory test results throughout the patient’s hospital stay. Arrows indicate high or low parameter levels.

Variable Tested	Hospital Days																			
	Emergency		Intensive Care Unit														Internal Medicine		Follow-Up	
Days	1		2a		2b		3a		3b		3c		4		5		6		38	
White blood cell count (G/L)	10.88	↑	8.85		10.01	↑	6.30		6.68				5.67		7.03		11.17	↑	10.52	↑
Neutrophil (%)	89.2	↑	92.9	↑	94.5	↑	82.9	↑	78.7	↑			82.3	↑	75.0	↑	78.6	↑	65.3	
Lymphocytes (%)	5.4	↓	2.7	↓	2.9	↓	4.9	↑	8.1	↓			6.0	↓	13.0	↓	8.2	↓	23.8	
Monocytes (%)	4.7		3.7		1.7	↓	6.0		7.1				6.8		9.9		11.6		5.8	
Red blood cell count (T/L)	5.73	↑	5.11		5.26		4.59		4.67				4.11	↓	4.09	↓	4.57		4.69	
Haemoglobin (g/L)	178	↑	158		176		143		146				129	↓	126	↓	142		146	
Haematocrit (%)	52.2		46.2		48		41.9		43.3				37.7	↓	37.2	↓	41.7		42.1	
MCV (Total cell count) (fL)	91.1		90.3		91.3		91.3		92.9				91.7		91.0		91.3		89.9	
MCH (Total cell count) (pg)	31.1		30.8		33.4	↑	31.2		31.3				31.3		30.9		31.1		31.1	
MCHC (Average Hgb conc.) (g/L)	341		341		366	↑	342		337				342		340		341		346	
Platelet count (G/L)	383		257		353		184		178				142	↓	141	↓	218		313	
Prothrombin activity (%)	0.68	↓	0.59	↓					0.83				0.88		0.85		0.77	↓	0.85	
PROTROMBIN (INR)	1.34	↑	1.51	↑	1.65	↑	1.25	↑	1.13				1.09		1.12		1.19		1.12	
Prothrombin time (s)	15.1	↑	17.1	↑	18.7	↑	14.1	↑	12.8				12.3		12.7		13.5		12.6	
APTI (s)									37.9	↑										
D-dimer (ng/mL)									949	↑										
Fibrinogen (g/L)					2.26															
Sodium (mmol/L)	141		143		140		141		140				143		140		139		140	
Potassium (mmol/L)	4.4		2.9	↓	3.4	↓	3.4	↓	3.7				3.5		4.2		4.9		4.5	
Calcium (mmol/L)			1.89	↓			1.86	↓	1.87	↓			1.86	↓	2.10	↓				
Glucose (mmol/L)	6.8		7.4	↑			6.7	↑	9.4	↑			7.4	↑	6.2	↑			5.1	
Bilirubin total (umol/L)	7.2		7.5				7.8		9.4				8.9		12.7		15.6		6.8	
Total protein (g/L)	81.5		61.5	↓													73.5		74.7	
Albumin (g/L)	46.4		35.5																	
Urea (mmol/L)	11.3	↑	9.6	↑			7.3		6.7				5.1		6.4		9.5	↑	7.3	
Creatinine (umol/L)	125.0	↑	102.0				82		87.0				70.0		60.0		93.0		107.0	
Glomerular filtration rate (mL/min/1.73 m^2^)	52	↓	65 2				84		78				101		120		72		62	
GOT (AST) (U/L)	94	↑	89	↑			90	↑	97	↑			81	↑	98	↑	95	↑	20	
GPT (ALT) (U/L)	45		39				41	↑	50				62	↑	118	↑	174	↑	25	
Gamma-GT (U/L)	33						24		27								192	↑	30	
LDH (U/L)	532	↑	385	↑			347	↑	376	↑			295	↑	313	↑	329	↑	182	
Amylase (U/L)	106	↑					101	↑	145	↑									93	
Lipase (U/L)	94	↑							218	↑										
CK (U/L)	295	↑							858	↑										
NT-proBNP (pg/mL)									2600	↑										
Cardiac Troponin T (ng/L)	30	↑	42	↑	31	↑	25	↑	29	↑	25	↑	37	↑						
hs-CRP (C-reactive protein) (mg/L)	8.9	↑	32.1	↑	31	↑	40	↑	35.6	↑			23.6	↑	17.1	↑	67.6	↑	1.0	
Procalcitonin (PCT) (ng/mL)			1.20	↑	0.47	↑	0.78	↑	0.64	↑			0.40		0.29					

**Table 2:** Blood gas analysis results throughout the patient’s hospital stay. pCO_2_: partial pressure of carbon dioxide (mmHg); pO_2_: partial pressure of oxygen (mmHg); HCO_3_^−^: standard bicarbonate (mmol/L); BE: base excess; Glu: blood glucose (mmol/L); Lac: lactate (mmol/L).

Day	1			2			3							4			5	
Hour	15:28	17:13	20:33	5:56	13:47	18:44	6:00	11:34	12:04	12:26	13:25	14:57	18:06	5:29	12:25	17:41	5:52	12:04
Sample Type	Venous	Arterial	Arterial	Arterial	Arterial	Arterial	Arterial	Arterial	Arterial	Arterial	Arterial	Arterial	Arterial	Arterial	Arterial	Arterial	Arterial	Arterial
pH	7.345	7.426	7.423	7.457	7.43	7.448	7.456	7.464	7.446	7.295	7.381	7.402	7.459	7.45	7.439	7.422	7.432	7.452
pCO_2_	42.9	36.1	36.7	38.7	39.9	37.1	37.4	33.9	33.9	52	43	43.2	37	39	37.1	39.1	39.1	38.1
pO_2_	37.8	58.5	57.1	52.7	50.3	52.5	57.2	54.5	48.6	35.1	119	109	63.3	66.4	65.7	75.2	58.2	62.1
HCO_3_^−^	21.7	24.1	24.2	27.1	25.9	25.7	26.5	25.1	24.1	21.9	24.6	26	26.5	26.9	25.3	25.3	25.8	26.6
BE	−2.2	−0.7	−0.4	3.4	2.1	1.6	2.5	0.5	−0.7	−1.2	0.4	2.1	2.4	3.1	0.9	1	1.8	2.7
Glu	6.3	5	6.2	7	5.1	5.7	6.4	6.4	6.5	8.7	7.8	8	7.6	7.5	9	8.6	6	6.3
Lac	1.7	0.9	0.8	0.8	0.8	0.7	0.7	0.8	1.7	2.1	0.7	0.6	0.5	1.2	1.5	1.5	1	0.7

**Table 3:** Fluid balance chart.

Day		1	2	3	4	5
Fluid Input (mL)	Infusion	4100	2880	3880	1440	0
	Oral intake	300	1700	1050	3000	1000
	Iv. drug admin.	512	764	732	654	200
	Total (mL)	4912	5344	5662	5094	1200
Fluid output (mL)	Urine	3500	2900	5445	4600	3400
	Perspiration (aprox.)	1000	1000	1000	1000	500
	Fecally excreted fluid	0	500	300	700	300
	Total (mL)	4500	4400	6745	6300	4200
Fluid balance (mL)		400	944	−1083	−1206	−3000
Cumulative fluid balance (mL)		400	1344	261	−945	−3945

## Data Availability

The original contributions presented in this study are included in the article. Further inquiries can be directed to the corresponding author.

## References

[1] Angelidis C., Kotsialou Z., Kossyvakis C., Vrettou A.-R., Zacharoulis A., Kolokathis F., Kekeris V., Giannopoulos G. (2018). Colchicine Pharmacokinetics and Mechanism of Action. Curr. Pharm. Des..

[2] Imazio M. (2015). Colchicine for pericarditis. Trends Cardiovasc. Med..

[3] Wu J., Liu Z. (2022). Progress in the management of acute colchicine poisoning in adults. Intern. Emerg. Med..

[4] Stack J., Ryan J., McCarthy G. (2015). Colchicine: New Insights to an Old Drug. Am. J. Ther..

[5] Nara T., Nakae H., Irie Y., Kameyama K., Okuyama M. (2023). Beware of accidental ingestion of *Colchicum autumnale* mistaken for *Allium victorialis*. Acute Med. Surg..

[6] Arif T., Bicker W., Pöchacker S., Kögler A., Gangl C., Holzer A. (2023). *Colchicum autumnale* intoxications: Experience of the Poisons Information Centre, Austria 2002–2018. Clin. Toxicol..

[7] Razinger G., Kozelj G., Gorjup V., Grenc D., Brvar M. (2021). Accidental poisoning with autumn crocus (*Colchicum autumnale*): A case series. Clin. Toxicol..

[8] Brvar M., Ploj T., Kozelj G., Mozina M., Noc M., Bunc M. (2004). Case report: Fatal poisoning with *Colchicum autumnale*. Crit. Care.

[9] Wollersen H., Erdmann F., Risse M., Dettmeyer R. (2009). Accidental fatal ingestion of colchicine-containing leaves–toxicological and histological findings. Leg. Med..

[10] Nagesh K., Menezes R.G., Rastogi P., Naik N., Rasquinha J.M., Senthilkumaran S., Fazil A. (2011). Suicidal plant poisoning with *Colchicum autumnale*. J. Forensic. Leg. Med..

[11] Finkelstein Y., Aks S.E., Hutson J.R., Juurlink D.N., Nguyen P., Dubnov-Raz G., Pollak U., Koren G., Bentur Y. (2010). Colchicine poisoning: The dark side of an ancient drug. Clin. Toxicol..

[12] (2017). ACMT Position Statement: Guidance for the Use of Intravenous Lipid Emulsion. J. Med. Toxicol..

[13] Nakatsu L., Lopez J.R., Garcia C.M., Cherian M., Nash J., Tofighi D., Seifert S.A., Smolinske S., Warrick B.J. (2025). Comparison of two-bag and three-bag acetylcysteine regimens in the treatment of paracetamol poisoning: A systematic review and meta-analysis. Clin. Toxicol..

[14] Güven A.G., Bahat E., Akman S., Artan R., Erol M. (2002). Late Diagnosis of Severe Colchicine Intoxication. Pediatrics.

[15] Mroz L. (2008). Variation of colchicine and metal content in *Colchicum autumnale* L. (Liliaceae) corms in relation to edaphic environment. Pol. J. Ecol..

[16] Alirezaie Noghondar M., Arouiee H., Shoor M., Shamsali R. (2013). Comparison of colchicine content between hysteranthous and synanthous *Colchicum* species in different seasons. Glob. J. Res. Med. Plants Indig. Med..

[17] Kupper J., Rentsch K., Mittelholzer A., Artho R., Meyer S., Kupferschmidt H., Naegeli H. (2010). A fatal case of autumn crocus (*Colchicum autumnale*) poisoning in a heifer: Confirmation by mass-spectrometric colchicine detection. J. Vet. Diagn. Investig..

[18] Hunter A.L., Klaassen C.D. (1975). Biliary excretion of colchicine. J. Pharmacol. Exp. Ther..

[19] Hendrickson R.G. (2019). What is the most appropriate dose of N-acetylcysteine after massive acetaminophen overdose?. Clin. Toxicol..

[20] Raghu G., Berk M., Campochiaro P.A., Jaeschke H., Marenzi G., Richeldi L., Wen F.-Q., Nicoletti F., Calverley P.M.A. (2021). The Multifaceted Therapeutic Role of N-Acetylcysteine (NAC) in Disorders Characterized by Oxidative Stress. Curr. Neuropharmacol..

[21] Iosfina I., Lan J., Chin C., Werb R., Levin A. (2012). Massive Colchicine Overdose with Recovery. Case Rep. Nephrol. Urol..

[22] Golob S., Zhang R.S., Medamana J.L., Pires K.D., Cruz J., Grossman J., Biary R., Divita M., Yuriditsky E. (2024). Colchicine Overdose: Challenges with Venoarterial Extracorporeal Membrane Oxygenation and Microaxial Flow Pump Support. JACC Case Rep..

[23] Sheibani M., Zamani N., Gerami A.H., Akhondi H., Hassanian-Moghaddam H. (2022). Clinical, Laboratory, and Electrocardiographic Findings in Colchicine Toxicity: 10 Years of Experience. Front. Med..

